# SPE-HPLC-DAD Dosage of Seven Neonicotinoids in Green Coffee

**DOI:** 10.3390/molecules30091930

**Published:** 2025-04-26

**Authors:** Serenella Seccia, Stefania Albrizio, Irene Dini

**Affiliations:** Department of Pharmacy, University of Naples Federico II, Via Domenico Montesano 49, 80131 Napoli, Italy; seccia@unina.it (S.S.); stefania.albrizio@unina.it (S.A.)

**Keywords:** food safety, pesticide analysis, SPE-HPLC/DAD, neonicotinoids, green coffee, chemical method validation

## Abstract

Green coffee is essential in many tropical economies. Its cultivation often necessitates using pesticides that can leave behind residues harmful to human health. To ensure consumer safety, the European Community has set strict maximum residue limits (ranging from 0.01 to 1.0 mg/kg) for pesticides in green coffee sold within Europe. However, the lack of official testing methods for neonicotinoids (NEOs) is a problem, as laboratories must spend resources and time developing and validating suitable analytical methods. This study developed and validated a method for the simultaneous analysis of seven NEOs frequently used in coffee cultivation: acetamiprid, clothianidin, dinotefuran, imidacloprid, nitenpyram, thiacloprid, and thiamethoxam. The proposed methodology uses Strata^®^-X PRO cartridges (solid-phase extraction) to remove interfering compounds present in the food matrix and high-performance liquid chromatography (HPLC), equipped with a diode array detector (DAD), to determine NEOs. The accuracy profile strategy validated the method’s suitability for the intended application. NEO recovery rates above 97%; negligible matrix effects (>93%); the linearity of the quantification method (R^2^ values above 0.99); relative biases and standard deviations below 5% and 6%, respectively; and an expected error rate less than 8% allowed to consider the method reliable for the intended objectives. Because of its low ecological impact and simple execution, this method can be used in routine analyses.

## 1. Introduction

**Figure 1:**
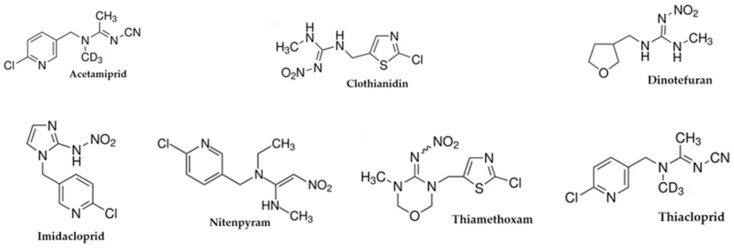
Neonicotinoids checked in green coffee samples.

Coffee is one of the most bought and sold commodities. Its remarkable export rates are responsible for the economic growth of tropical agricultural regions [1]. Green coffee seeds are the raw *Coffea* plant’s (*Rubiaceae* family) seeds [2]. They can be roasted to create the beloved beverage or utilized to isolate compounds such as chlorogenic, quinic, p-coumaric, and caffeic acids, which are used for manufacturing weight-loss supplements [3,4,5]. The Brazilian Ministry of Agriculture permits the use of pesticides in coffee farming, including neonicotinoids, carbamates, diamides, and organophosphates, to control phytophagous and fungal pathogens that pose a serious threat to plant health and reduce the seeds’ yield [6,7,8]. However, they can negatively affect food security. Neonicotinoids act as endocrine disruptor chemicals (EDCs) [9,10]. Recent epidemiology, in vivo, and in vitro research has highlighted significant potential risks of neonicotinoid exposure. These substances can adversely affect mammalian sperm fertility and embryonic development. They can have genotoxic effects that lead to DNA damage. Furthermore, neonicotinoids can activate acetylcholine receptors and negatively affect the liver enzymes’ synthesis [11]. Specifically, the neonicotinoid imidacloprid can promote adipogenesis and insulin resistance. Exposure to nitenpyram during pregnancy can alter gut microbiota composition and cause nonalcoholic steatohepatitis. Perinatal exposure to clothianidin has been associated with reproductive toxicity, while exposure to acetamiprid may negatively affect memory consolidation processes [9]. To safeguard consumers’ health, the European Community has imposed maximum residue limits for pesticide levels in green coffee marketed within Europe. These limits are set between 0.01 and 1.0 milligrams per kilogram [12]. The absence of official testing methods for neonicotinoids (NEOs) presents a significant challenge. Various methods have been proposed for dosing NEOs in raw green coffee [13,14,15,16,17,18,19,20,21], all involving an initial cleanup step to remove matrix interferents before proceeding with NEO dosage. The most commonly used method is QuEChERS (quick, easy, cheap, effective, rugged, and safe), which consists of two steps: extraction with MgSO_4_ and acetonitrile to remove water from the organic phase and purification with dispersive solid-phase extraction (d-SPE). The purification process utilizes primary and secondary amine (PSA-SPE columns) to eliminate polar organic acids, pigments, fatty acids, and sugars, eventually followed by graphitized carbon black to eliminate sterols and chlorophyll or C18 to eliminate nonpolar interfering substances such as lipids [22,23,24,25,26,27]. NEO dosage, instead, is performed with sophisticated and expensive equipment, such as liquid chromatography (generally C-18 reverse phase) coupled with mass spectrometry (LC-MS) [28] or gas chromatography coupled with mass spectrometry (GC-MS) [13,15]. Unfortunately, mass spectrometers are not in most laboratories that perform routine food analysis because of their high purchase and maintenance costs. Furthermore, complex pesticide extraction processes are required to reduce interference from other substances, such as caffeine, when analyzing neonicotinoids with GC-MS. As a result, the costs and time involved in these analyses make many of the methods described in the literature impractical for routine use [19,20]. This study suggests a simple method for the simultaneous quantification of seven NEOs, acetamiprid (ACT), clothianidin (CLT), dinotefuran (DNT), imidacloprid (IMD), thiacloprid (TCP), nitenpyram (NTP), and thiamethoxam (THT), in green coffee samples (Figure 1).

The method was developed to be compatible with equipment typically found in commodity laboratories and adhere to standard analysis times. It includes a user-friendly cleanup process using Strata^®^-X PRO (Phenomenex (Torrance, CA, USA)) cartridges that can efficiently remove interfering substances in just one step, requiring no special operating skills, followed by HPLC-DAD dosage. The method was validated to ensure the results’ traceability and reliability using the accuracy profile strategy proposed by the Société Française des Sciences et Techniques Pharmaceutiques Commission [29,30,31,32]. This validation approach has been previously positively applied to dose other pesticides such as glyphosate and glufosinate in many foods [33], aflatoxins in almonds [34], furan in apple puree and infant formula [35], and neonicotinoids in wheat [36] and Moroccan spearmint [37].

## 2. Results

The method was designed to provide highly efficient analysis while remaining cost-effective. Validation sample concentrations were back-calculated to assess relative bias, repeatability, intermediate precision, and β-expectation tolerance intervals at the 95% probability level.

### 2.1. Extraction and Cleanup Procedure

**Figure 2:**
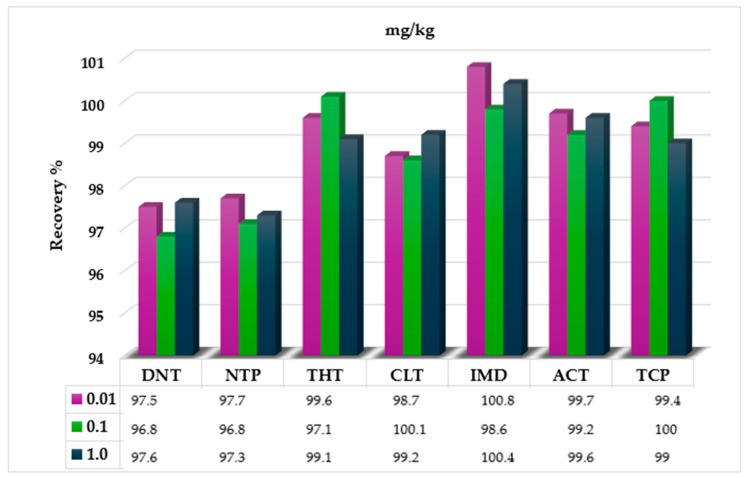
Recovery values (%) of the seven neonicotinoids (0.01, 0.1, and 1.0 mg/kg) obtained with the mixture of dichloromethane/methanol (9:1) in green coffee samples.

NEO-free green coffee samples were introduced into STRATA XPRO cartridges without prior conditioning or equilibration. Various solvents and mixtures were tested to identify the optimal mobile phase for maximizing NEO recovery. The best results were achieved with a dichloromethane/methanol mixture (9:1, *v*/*v*). NEO recovery rates ranging from 97.1% to 101.0% and minimal matrix interference proved the high extraction efficiency of the mixture (Figure 2).

### 2.2. Selectivity and Carryover Evaluation

**Figure 3:**
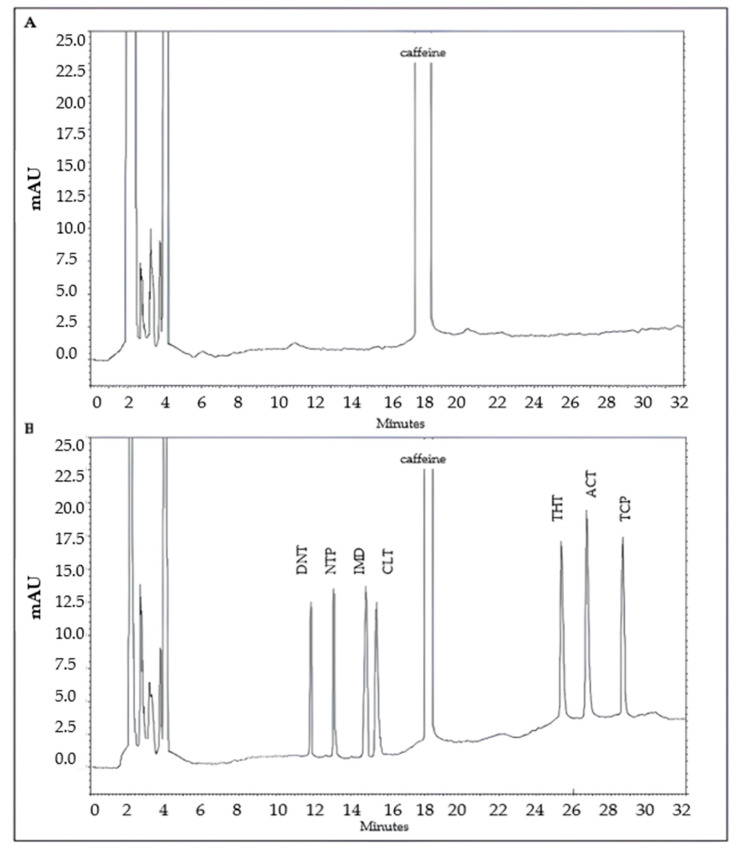
(**A**) HPLD-DAD chromatogram after extraction with STRATA XPRO cartridge of a blank green coffee sample; (**B**) chromatogram of a green coffee sample enriched with 0.1 mg/kg of a multicomponent solution containing the seven neonicotinoids examined (black line), including the caffeine peak.

The test selectivity was validated by comparison of the retention time and UV spectra of each peak to standards [38]. Eight peaks were observed, of which seven were ascribed to NEOs (dinotefuran, Rt 11.8 min; nitenpyram, Rt 13.2 min; imidacloprid, Rt 14.9 min; clothianidin, Rt 15.5 min; thiamethoxam, Rt 25.3 min; acetamiprid, Rt 26.7 min; thiacloprid, Rt 28.8 min) and one was associated with caffeine (Rt 8.2 min) (Figure 3).

The chromatographic accuracy of quantitative analysis was evaluated by determining carryover, which can happen when a high concentration of an analyte remains on the column and influences the quantification of later injections. The NEO carryovers were: 1.41% (SD = 0.25%, *n* = 3) for acetamiprid; 1.62% (SD = 0.30%, n = 3) for clothianidin; 1.33% (SD = 0.14%, n = 3) for dinotefuran; 1.71% (SD = 0.74%, n = 3) for imidacloprid; 1.87% (SD= 0.34%, n = 3) for thiacloprid; 1.51% (SD = 0.28%, n = 3) for nitenpyram; and 1.91% (SD = 0.58%, n = 3) for thiamethoxam. All results complied with the acceptance standards (<5% for the internal standard) specified in the EMA Guideline for Process Validation. The chromatographic reliability was also evaluated by determining the peak symmetry. The coefficient of peak symmetry was used to determine peak asymmetry. Peak symmetry describes how closely the shape of a chromatographic peak aligns with a Gaussian (bell-shaped) curve. It plays a vital role in ensuring the accuracy and precision of quantitative analyses. The seven NEO asymmetry factors ranged from 0.81 to 1.2. According to the United States Pharmacopeia, optimal peaks should have an asymmetry factor of 1, although values ranging from 0.8 to 1.8 are considered acceptable [39].

### 2.3. Linearity

The method linearity refers to its capacity to give outcomes directly proportional to the analyte concentration within a defined range. The method’s linearity was measured across five calibration levels. Solvent calibration and matched-matrix calibration curves were developed. The solvent calibration solutions were obtained by adding NEO standards to the solvent. Matched-matrix calibration solutions were created by incorporating known levels of the NEOs working solutions into blank sample extracts [40]. The calibration curves demonstrated the linear relationship between the theoretical and measured concentrations (for each NEO, R^2^ > 0.999; Table 1).

### 2.4. Matrix Effect

The matrix effect was evaluated using standards in the chromatographic mobile phase that were compared with matrix-matched standards. The matrix effects were quantified as relative standard deviations (%).

(1) $$\mathrm{ME}\;(\%)=\frac{Slope\;solvent}{Slope\;matrix}\;\times 100$$

The matrix effect for each NEO (>93%) demonstrated that it was not very important when determining the target compounds (Table 1).

### 2.5. Trueness

Trueness serves as an indicator of the method’s systematic errors. It reveals how closely the experimental data align with the accepted reference values. The relative bias percentage, evaluated at three fortification levels, was calculated as the difference percentage between the reference value and the experimental results average. The excellent method’s trueness was proven by relative bias (%) at each concentration level less than 3.2% (Table 2).

### 2.6. Precision

Precision serves as an indicator of the method’s random error. It was assessed by examining the intraday and interday precision across various concentration levels (0.01, 0.1, and 1.0 mg/Kg). The results were expressed as relative standard deviation (RSD %) [41].

(2) $$\mathrm{RSD}\%=\frac{S*100}{\bar{x}}$$

The method demonstrated excellent reproducibility in short and extended periods, as indicated by RSD% values between 1.1 and 4.1 (Table 2).

### 2.7. Limits of Detection (LOD) and Quantification (LOQ)

The values of LOD and LOQ (Table 3) were calculated from ordinary least-squares regression data, as indicated in Mancusi et al. [42].

In this work, the lowest sample concentration at which we could achieve acceptable assay precision and accuracy was always below the maximum residue limit (MRL) established for each NEO in coffee by the European food safety legislation [12].

### 2.8. Accuracy

**Figure 4:**
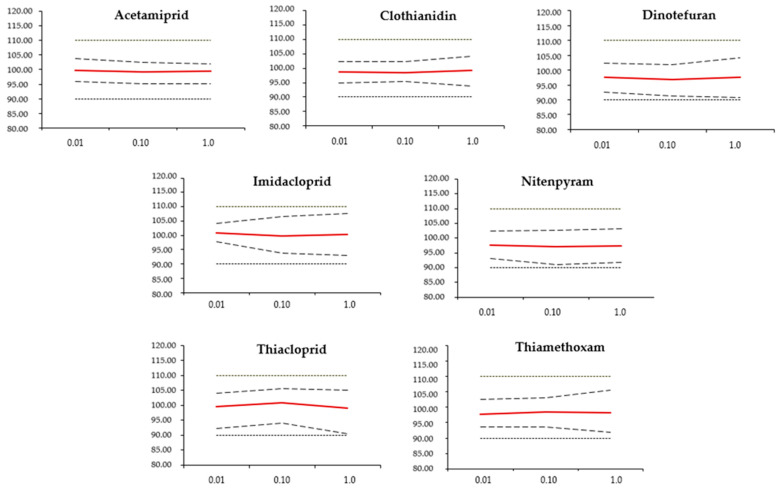
Accuracy profiles of the seven extracted neonicotinoids. The x-axis represents concentrations (mg/kg), while the y-axis indicates a recovery percentage. The dotted lines are the ±10% acceptance limits; the dashed lines are the upper and the lower 95% expectation tolerance limits; the red lines are the recovery yield (%).

Accuracy considers the total error, the sum of systematic and random errors, in the test results. It reflects how closely the test results agree with the acceptance reference value [43]. The accuracy profile for each neonicotinoid insecticide is reported in Figure 4. The analytical method was deemed valid within the defined dosing range, as the β-expectation tolerance interval (dashed lines) did not exceed the acceptance limits (dotted lines) for total error at each concentration level, confirming the method’s accuracy. As shown in Figure 4, the β-expectation tolerance intervals (Table 2) remained within the acceptance limits across the entire dosing range assessed. This guaranteed that the outcomes of later analyses would go beyond an error of ±10%.

### 2.9. Uncertainty

Uncertainty refers to the range of values that can reasonably be attributed to the analyte. In this study, the extended uncertainty was defined as the interval within which the unknown “true” value can be observed with a 95% confidence level. The relative expanded uncertainty (%) was determined by dividing the expanded uncertainty by the corresponding concentration and applying a coverage factor of k = 2, representing a 95% confidence interval within which the true value was expected to lie. The calculated relative expanded uncertainties did not exceed 8%. (Table 2). Based on the relative expanded uncertainty, Table 2 reports the acceptable range of concentration values (mg/kg) for each NEO.

## 3. Discussion

This study developed and validated a reliable, straightforward method for simultaneously quantifying seven NEOs in green coffee. Notably, a Strata-X PRO cartridge was utilized for the first time to concentrate the NEOs. Usually, the QuEChERS method is used to optimize the extraction of NEOs from food matrices. This method is recognized for its straightforwardness, efficiency, and effectiveness. Some criticisms and limitations of the QuEChERS method include variable recoveries, salt precipitation, inadequate removal of interferences, and the need for additional cleanup steps for specific complex samples [19,44]. Strata-X PRO cartridges are filled with an advanced polymeric SPE sorbent using matrix removal technology. These cartridges extract the analytes in just two steps (sample loading and rinsing). The Strata-X PRO method is quicker than the QuEChERS approach and minimizes the chances of losing NEOs, as it consists of a single extraction step [36]. Thus, these cartridges are more efficient and labor-saving than the traditional QuEChERS cleanup method. As indicated by earlier studies, the quantification process was performed using high-performance liquid chromatography equipped with a diode array detector and a C18 column [45]. Earlier studies suggested two types of detectors for this analysis: the DAD spectrophotometer and the mass spectrometer. The DAD detector is typically less selective and sensitive than a mass spectrometer but presents the notable benefit of lower acquisition and maintenance expenses. This cost-effectiveness enhances its accessibility and availability in standard laboratories, fostering its widespread use for routine analyses. Among the C-18 columns available, the Kinetex C18 column, which features advanced core-shell technology, was chosen as the stationary phase to reduce band broadening during the separation process. The chromatographic conditions were adjusted to achieve the NEO elution in a short analysis time. Eight distinct peaks were identified. Seven peaks were associated with the NEOs, and one peak was identified as caffeine, following a comparison with both the retention times and UV spectra of the analytical standards. Caffeine did not influence the NEOs’ determination, as retention time did not coincide. The peak asymmetry factors being within acceptable limits and the absence of residual peaks from previous injections (no remarkable carryover effect) excluded any interference with the NEO dosage, confirming that the chromatographic system was suitable for its intended use. Generally, metrological validation methods for pesticide analysis emphasize matrix effects, linearity, bias, precision, and uncertainty, with a strong dependence on null hypothesis testing. These methods prioritize the risks associated with suppliers, neglecting the important equilibrium between consumer safety and the risks faced by producers. It is crucial to evaluate uncertainty to overcome this limitation and fulfill the requirements for ISO 17025 compliance [37]. For this purpose, the Société Française des Sciences et Techniques Pharmaceutiques (SFSTP), aligning with ICH Q2 guidelines (2022), introduced the “accuracy profile approach” [46,47]. In contrast to other metrological approaches, the “accuracy profile approach” offers a distinct advantage by addressing the risks tied to the method’s future applications rather than depending solely on predefined acceptance limits. It also evaluates the probability of measurements falling outside the desired range during routine analyses, instilling confidence in analysts that the method will consistently deliver accurate results. The validation process confirmed that the instrumentation effectively detected NEO levels below the maximum tolerable thresholds set by Commission Regulation (EU) 2023/915 [48], with precision within acceptable limits for total error across all concentration levels, and results from future analyses are expected to consistently remain within a ±10% variation from the anticipated values.

## 4. Materials and Methods

### 4.1. Solvents and Reagents

Acetonitrile, dichloromethane, methanol, and acetic acid (all HPLC grade) were acquired from Carlo Erba Reagents (Carlo Erba Reagents, Cornaredo, MI, Italy).

Water for HPLC was collected by distilling and vacuum filtering water on Millipore filters (Millipore HA WP 04700; Burlington, MA, USA).

Strata^®^-X PRO cartridges were bought by Phenomenex (Torrance, CA, USA)

### 4.2. Analytical Standards

Acetamiprid, clothianidin, dinotefuran, imidacloprid, nitenpyram, thiacloprid, and thiamethoxam (PESTANAL^®^, analytical standard), as well as caffeine (purity ≥ 99%) were supplied from Sigma-Aldrich (St. Louis, MI, USA).

### 4.3. Samples

The Chemical Laboratory of the Naples Customs and Monopolies Agency generously provided the green coffee bean samples. The seeds were ground by employing a coffee grinder (Model No. 560.01, Capresso, Montvale, NJ, USA) and sieved by using a sieve with mesh ASTM10 (Model: V8SF #10, Gilson, Lewis Center, OH, USA) to obtain a sample with a diameter of 2 mm. The processed samples were stored in glass bottles at 4 °C, and all analyses were conducted in triplicate.

### 4.4. Sample Extraction and Cleanup with Strata-X PRO Cartridge

A 1 g aliquot of green coffee sample, found negative for the target insecticides, was placed into a 50 mL polypropylene conical tube. It was then soaked with 6 mL of cooled water for 20 min, and 4 mL of acetonitrile (ACN) was added to it. The mixture was vortexed for 2 min and allowed to rest for 5 min before being centrifuged at 5000× *g* for 20 min at room temperature. The supernatant was collected and slowly poured onto a Strata-X PRO cartridge. The cartridge was then dried under full vacuum for 1 min. The samples were eluted gradually with 20 mL (5 × 4 mL) of dichloromethane containing 10% methanol. The effluents were collected and concentrated using a rotary vacuum evaporator. The resulting dried extract was reconstituted with 1 mL of the initial chromatographic mobile phase and analyzed by HPLC-DAD.

### 4.5. HPLC (High-Performance Liquid Chromatography) Parameters

LC experiments were conducted using an Agilent Technologies 1200 series system equipped with a diode array detector set to a wavelength of 260 nm. Data were acquired and analyzed using the standard Agilent ChemStation^®^ software Rev. B.02.01 (Agilent, Palo Alto, CA, USA). The analytical column used was a Kinetex C18 (150 mm × 4.6 mm, μm particle size) from Phenomenex (Torrance, CA, USA), coupled with a Phenomenex C18 guard cartridge (4 × 8 mm) at a temperature of 25 °C. The injection volume was 20 μL, and the flow rate was 1.0 mL/min. The UV wavelength used in the experiment was 260 nm. The mobile phase consisted of water with 0.2% formic acid (A) and acetonitrile (B), and the insecticides were separated using the following LC gradient program: 0–5 min, 10% B; 5–10 min, 25% B; 10–15 min, 35% B; 15–20 min, 50% B; 20–30 min, 60% B; 31–40 min, return to 5% B for system equilibration. NEOs were identified by their retention times, and quantification was performed using the peak area ratio of the target analytes compared with an external standard. Spiked blank samples were used as standards to counteract any potential matrix effects, with the sample being spiked before extraction. The asymmetry factor was employed to estimate the chromatographic separation.

$$\mathrm{Asymmetric}\;\mathrm{factor}=\frac{b}{a}$$

*b* and *a* were evaluated at 10% of the maximum peak’s height; *b* represents the length from the peak’s highest point to its trailing edge, while a represents the distance from the front edge of the peak to its highest point [49].

The carryover effect was assessed to confirm no residual peaks from previous injections. It was assessed by injecting the blank samples following a fortified sample at a 2 mg/mL concentration [50].

### 4.6. Method Validation

Method validation was carried out using the total error approach, based on the β-expectation tolerance interval recommended by the Society of Pharmaceutical Science and Techniques Commission (SFSTP), with an acceptability limit of λ ± 10%. The β-expectation tolerance interval was 95% [29,30,31]. The expanded uncertainty was calculated using a coverage factor of k = 2, indicating an interval around the results within which the true unknown value can be identified with a 95% confidence level [31].

#### 4.6.1. Experimental Designs

The designs for calibration and validation involved a duration of 3 days (k = 3), with 3 replicates (n = 3) and 5 concentration levels (m = 5) for the calibration standards, along with 3 concentration levels (m = 3) for the validation standards. Measurements for both validation and calibration were gathered on the same days.

#### 4.6.2. Calibration Standards

A stock solution of each NEO (1 mg/mL) was obtained by mixing 50 mg of each NEO in 50 mL of acetonitrile. A 5-point calibration curve was prepared for each experiment (0.01, 0.05, 0.1, 0.5, and 1.0 µg/mL). Calibration standards were prepared by diluting a standard multicomponent solution containing the seven NEOs (10 µg/mL) to achieve final concentrations of 0.01, 0.05, 0.1, 0.5, and 1.0 µg/mL. Matrix-matched calibration curves were developed to assess how interfering compounds impact the quantification of NEOs using blank matrix extracts at identical concentration levels.

#### 4.6.3. Validation Standards

Validation samples were prepared by spiking blank matrix samples, resulting in negative results in the analysis, to achieve final validation standards at three fortification levels corresponding to spike concentrations of 0.01, 0.1, and 1.0 mg/kg. The linear relationship between theoretical and measured concentrations and relative bias, repeatability, intermediate precision, and β-expectation tolerance intervals (at the 95% probability level) were assessed using the calibration curve.

### 4.7. Statistical Analysis

Microsoft Excel 2010 (Microsoft, Redmond, WA, USA) was employed for statistical analyses.

## 5. Conclusions

A straightforward and efficient HPLC-DAD method was developed and validated to determine the levels of seven neonicotinoid insecticides in green coffee simultaneously. This method involved a cleanup procedure on Strata-X PRO cartridges, the separation of each NEO using a C18 Kinetex column, and spectrophotometric NEO quantification with DAD. The Strata^®^-X PRO cartridge is a groundbreaking SPE tool that effectively eliminates interfering substances in one step with minimal solvent usage. This solution offers substantially lower cost and shorter analysis durations than the QuEChERS cleanup methods usually employed for this purpose. Additionally, the financial burden of purchasing and sustaining DAD spectrophotometer systems as HPLC detectors is significantly lower than that of purchasing and sustaining the mass spectrometers suggested in previous methods. The effectiveness of the method for its intended application was confirmed through high recovery rates and the absence of interfering substances at the retention time of the NEOs, demonstrating excellent extraction efficiency and a distinct and consistent linear relationship between the independent and dependent variables, along with negligible differences in both short- and long-term precision that showcased low error margins in the quantification method, underscoring its reliability and accuracy. Thus, the suggested test is well-suited for regular official assessments, since it can accurately and precisely quantify the NEO levels allowed by existing regulations in green coffee using technologies commonly found in commodity laboratories and simple and environmentally friendly procedures. Future research should prioritize the development of robust methods for accurately dosing NEOs across food matrices to ensure food safety and human health.

## Figures and Tables

**Table 1:** Parameters derived from calibration curves of solvent standards and matrix-matched standards.

NEO		Slope	Intercept	R^2^	Residual (%)	Matrix Effect
DNT	Solvent Matrix	216.78 218.12	0.52 0.48	0.9996 0.9991	±0.2 ±2.2	99.38
NTP	Solvent Matrix	154.44 163.820	0.20 0.16	0.9997 0.9991	±1.7 ±1.9	94.27
THT	Solvent Matrix	150.930 159.940	−0.23 −0.04	0.9998 0.9999	±3.3 ±5.2	94.36
CLT	Solvent Matrix	176.400 188.950	−0.03 −0.44	0.9998 0.9996	±2.1 ±3.5	93.35
IMD	Solvent Matrix	189.57 197.25	0.69 0.83	0.9994 0.9993	±0.6 ±2.8	96.1
ACT	Solvent Matrix	216,78 222.95	0.55 0.61	0.9995 0.9991	±2.7 ±2.9	97.23
TCP	Solvent Matrix	180.12 185.50	−0.24 −0.05	0.9997 0.9995	±1.5 ±1.9	97.09

**Table 2:** Validation parameters for the seven neonicotinoids in green coffee.

Neonicotinoid	Concentration Level (mg/kg)	Relative Bias (%)	Intra- Assay Precision (RSD%)	Interassay Precision (RSD%)	β-Expectation Tolerance Limits (%)	Relative Expanded Uncertainty (%)	Range of Concentration Values (mg/kg)
Acetamiprid	0.01	−0.3	1.1	1.7	[−3.8;4.2]	2.3	0.0098/0.0102
	0.10	−0.8	2.4	2.0	[−4.0;3.4]	3.2	0.068/0.103
	1.00	−0.4	1.9	2.7	[−4.5;2.4]	2.6	0.97/1.03
Clothianidin	0.01	−1.3	2.0	4.5	[−4.0;3.7]	5.8	0.0094/0.0106
	0.10	−1.4	2.7	3.9	[−3.1;3.6]	4.7	0.095/0.105
	1.00	−0.8	2.6	4.4	[−5.3;5.1]	6.1	0.94/1.06
Dinotefuran	0.01	−2.5	2.6	5.2	[−5.0;4.9]	6.3	0.0094/0.0106
	0.10	−3.2	3.0	4.6	[−5.5;5.1]	5.5	0.094/0.105
	1.00	−2.4	4.8	5.9	[−6.9;6.6]	7.7	0.92/1.08
Imidacloprid	0.01	0.8	1.7	2.5	[−3.0;3.5]	3.1	0.0097/0.013
	0.10	−0.2	2.2	2.4	[−6.1;6.7]	3.3	0.097/0.103
	1.00	0.4	1.4	2.0	[−7.4;7.2]	3.6	0.96/1.04
Nitenpyram	0.01	−2.3	3.3	3.3	[−4.5;4.7]	7.9	0.0092/0.0108
	0.10	−2.9	3.2	3.9	[−6.1;5.6]	4.2	0.096/0.104
	1.00	−2.7	3.9	4.1	[−5.6;5.8]	6.4	0.94/1.06
Thiacloprid	0.01	−0.4	1.6	3.8	[−7.2;4.4]	4.8	0.0095/0.0105
	0.10	0.1	1.8	4.4	[−7.0;4.6]	5.9	0.094/0.106
	1.00	−1.0	2.7	3.6	[−8.4;6.0]	5.4	0.94/1.06
Thiamethoxam	0.01	−2.2	3.1	3.1	[−4.2;4.8]	5.4	0.0095/0.0105
	0.10	−1.5	4.1	4.2	[−5.0;4.6]	5.9	0.094/0.106
	1.00	−1.8	3.3	4.0	[−6.4;7.3]	6.1	0.95/1.05

**Table 3:** MRLs of NEOs in coffee and LODs and LOQs of NEOs identified using the suggested method.

NEO	LOD (mg/kg)	LOQ (mg/kg)	MRL (mg/kg)
DNT	0.003	0.01	0.01 (default MRL)
NTP	0.003	0.01	0.01 (default MRL)
THT	0.006	0.02	0.2
CLT	0.009	0.03	0.05
IMD	0.003	0.01	1.0
ACT	0.009	0.03	0.05
TCP	0.006	0.02	0.05

## Data Availability

Data is contained within the article.
